# SNP Discovery and Genetic Variation of Candidate Genes Relevant to Heat Tolerance and Agronomic Traits in Natural Populations of Sand Rice (*Agriophyllum squarrosum*)

**DOI:** 10.3389/fpls.2017.00536

**Published:** 2017-04-07

**Authors:** Pengshan Zhao, Jiwei Zhang, Chaoju Qian, Qin Zhou, Xin Zhao, Guoxiong Chen, Xiao-Fei Ma

**Affiliations:** ^1^Key Laboratory of Stress Physiology and Ecology in Cold and Arid Regions, Northwest Institute of Eco-Environment and Resources, Chinese Academy of SciencesLanzhou, China; ^2^Shapotou Desert Research and Experiment Station, Northwest Institute of Eco-Environment and Resources, Chinese Academy of SciencesLanzhou, China

**Keywords:** sand rice, physiological adaptation, climate change, single nucleotide polymorphism, allele diversity, natural variation, candidate genes

## Abstract

The extreme stress tolerance and high nutritional value of sand rice (*Agriophyllum squarrosum*) make it attractive for use as an alternative crop in response to concerns about ongoing climate change and future food security. However, a lack of genetic information hinders understanding of the mechanisms underpinning the morphological and physiological adaptations of sand rice. In the present study, we sequenced and analyzed the transcriptomes of two individuals representing semi-arid [Naiman (NM)] and arid [Shapotou (SPT)] sand rice genotypes. A total of 105,868 pairwise single nucleotide polymorphisms (SNPs) distributed in 24,712 Unigenes were identified among SPT and NM samples; the average SNP frequency was 0.3% (one SNP per 333 base pair). Characterization of gene annotation demonstrated that variations in genes involved in DNA recombination were associated with the survival of the NM population in the semi-arid environment. A set of genes predicted to be relevant to heat stress response and agronomic traits was functionally annotated using the accumulated knowledge from *Arabidopsis* and several crop plants, including rice, barley, maize, and sorghum. Four candidate genes related to heat tolerance (heat-shock transcription factor, *HsfA1d*), seed size (DA1-Related, *DAR1*), and flowering (early flowering 3, *ELF3* and *late elongated hypocotyl, LHY*) were subjected to analysis of the genetic diversity in 10 natural populations, representing the core germplasm resource across the area of sand rice distribution in China. Only one SNP was detected in each of *HsfA1d* and *DAR1*, among 60 genotypes, with two in *ELF3* and four in *LHY*. Nucleotide diversity ranged from 0.00032 to 0.00118. Haplotype analysis indicated that the NM population carried a specific allele for all four genes, suggesting that divergence has occurred between NM and other populations. These four genes could be further analyzed to determine whether they are associated with phenotype variation and identify alleles favorable for sand rice breeding.

## Introduction

Ongoing climate change and the increasing global population are continuous threats to global food security (Tester and Langridge, 2010; Lobell et al., 2011; Lobell and Gourdji, 2012; McCouch et al., 2013; Wheeler and von Braun, 2013). The negative influence of climate change on crop production has motivated scientists improve staple crops by exploitation of the genetic resource available in their wild relatives (Tester and Langridge, 2010; McCouch et al., 2013); however, the simultaneous development of new crops among the neglected and underutilized species will also crucial for sustainable and intensified food production (Mayes et al., 2012; Chen et al., 2014; Zhao et al., 2014). The Amaranthaceae species, sand rice (*Agriophyllum squarrosum*), has been among crops used for army provisions since the Tang Dynasty (AD 618–907) and is still an important component of local food for people inhabiting the Hexi Corridor along the ancient Silk Road in the northwest of China (Gao, 2002; Chen et al., 2014). Due to its high nutritional value and extreme stress tolerance, sand rice represents a suitable alternative food crop, resilient to climate change (Chen et al., 2014; Zhao et al., 2014).

Sand rice originates from the Gurbantunggut desert and is widely distributed on the mobile and semi-mobile sand dunes across the arid regions of northern China (Zhao et al., 2014; Qian et al., 2016). To thrive in a desert environment, sand rice has evolved many morphological traits and adaptation strategies to mitigate the risk of extinction due to climate variability, such as extreme temperatures, unpredictable precipitation, strong solar radiation, and other environmental stresses (Chen et al., 2014; Zhao et al., 2014). Comparative transcriptome analysis has been used to identify candidate genes related to abiotic stress tolerance and unique traits of this species (Zhao et al., 2014, 2016). Some core genetic elements involved in environmental responses are conserved among plant species; however, the genetic mechanisms underpinning the morphological and physiological adaptations of sand rice remain poorly understood.

Natural variation is the genetic basis for species adaptation to different environments and the identification of genomic polymorphisms, for example single nucleotide polymorphisms (SNPs), is essential for in-depth analysis of genes and alleles involved in plant evolution and environmental adaptation (Mitchell-Olds and Schmitt, 2006; Alonso-Blanco et al., 2009; Lasky et al., 2012). Extensive whole genome SNP analysis of several model and crop plants, such as *Arabidopsis thaliana*, rice, and maize, has been performed during past decades for a variety of purposes, including studies of genetic diversity, genome evolution, association mapping, and domestication (McNally et al., 2009; Huang et al., 2010; Lai et al., 2010; Hancock et al., 2011; Kump et al., 2011; van Heerwaarden et al., 2011; Horton et al., 2012).

**FIGURE 1:**
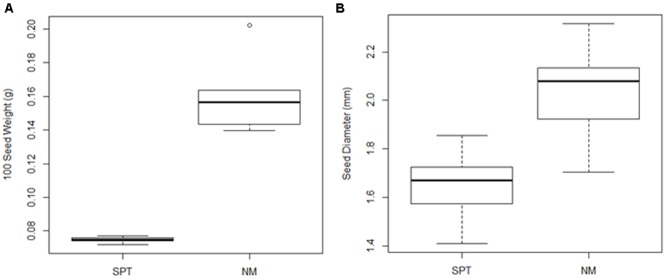
**Boxplots of seed weight and seed diameter of sand rice plants from two natural populations (SPT and NM). (A)** Seed weight was calculated using 100 seeds from each of five independent plants from each population; **(B)** Diameters of 10 seeds per plant from five independent plants were measured from both populations.

The climate varies considerably across the geographic range of sand rice, and precipitation and mean temperature of the coldest quarter strongly influence its distribution (Qian et al., 2016). Furthermore, phenology variation has been observed among natural populations of sand rice (Zhao et al., 2014; Yin et al., 2016); for example, the seed size from semi-arid region (Naiman, NM) is larger than that from arid region [Shapotou (SPT); **Figure 1**]. Moreover, the flowering of NM sand rice occurs earlier than that of SPT at the common garden; SPT individuals are more tolerant to heat stress. Large scale analyses of genetic variation will be crucial for understanding the genetic mechanisms underlying adaptation of sand rice to climate features or local environmental conditions, and provide a foundation for the discovery and isolation of markers useful for its subsequent domestication.

Previous studies in *Arabidopsis* have elucidated complex pathways regulating heat stress response, seed size, and flowering time (Fornara et al., 2010; Bokszczanin et al., 2013; Verhage et al., 2014; Li and Li, 2016). For example, the heat-shock transcription factor HsfA1d is one of master regulators with a critical role in evoking the transcription cascade to confer heat stress response (Ohama et al., 2016). The ubiquitin receptors, DA1 and DA1-related protein (DAR1), are core genetic elements controlling cell proliferation in the integument via the ubiquitin-proteasome pathway (Li and Li, 2016). Moreover, *late elongated hypocotyl* (*LHY*) and *early flowering 3* (*ELF3*) are morning- and evening-phased components involved in circadian rhythm regulation in *Arabidopsis* (Hicks et al., 1996; Nusinow et al., 2011; Hsu and Harmer, 2014).

With the advancement of sequencing technologies, genome scale analyses of sequence polymorphism has become feasible and cost effective for non-model plants (Silva-Junior et al., 2011; Zou et al., 2014; Pavy et al., 2016). Analysis of the genomes of populations from different environments could identify genes and alleles favored in specific local climates (Henry, 2014). In this study, two representative sand rice plants from arid and semi-arid regions (SPT and NM) were sequenced using the RNA-seq method to generate a genome wide dataset of SNP polymorphisms. Four candidate genes, *HsfA1d, DAR1, LHY*, and *ELF3*, were selected for further assessment of allelic diversity in 10 natural populations, which represent the core germplasm resources of sand rice, according to the results of phylogeographic analysis (Qian et al., 2016).

## Materials and Methods

### Ethics Statement

Shapotou and NM seeds were collected from SPT Desert Research and Experimental Station and NM Desertification Research Station, Northwest Institute of Eco-Environment and Resources, Chinese Academy of Sciences. Specific permits were not required to sample the seeds for this study.

### Seed Size

Seeds were sampled from five independent plants at each of the NM and SPT stations. For each plant, 100 seeds were used to calculate the average seed weight and 10 seeds were used to determine the average diameter.

### Plant Materials, RNA Extraction, Transcriptome Sequencing, and Unigene Annotation

Naiman seeds were germinated in a growth chamber and then transferred into pots filled with nutritional soil in the base with an upper layer of sand in a green house. The shoot and root of a 3-month-old seedling (Supplementary Figure 1) were collected separately and total RNA was extracted using a Plant total RNA Kit (TIANGEN, Beijing, China). Equal amounts of RNA from shoot and root were mixed together for cDNA library construction. Library was prepared as described by Zhao et al. (2014) and transcriptome sequencing was performed on the Illumina HiSeqTM 2500 platform using 125 bp paired-end reads at Biomarker Technologies (Beijing, China). A total of 16.40 million reads were obtained with 93.01% achieving quality scores above Q30. Raw data were deposited in the NCBI Short Read Archive with the accession number SRR5271162.

Reads from a previous transcriptome analysis of plant from the SPT region were downloaded from the NCBI Sequence Read Archive (SRR1559276, Zhao et al., 2014). After filtering, high quality reads from the two samples were assembled together by Trinity program with default settings (Grabherr et al., 2011). Contigs were clustered based on sequence similarity and paired-end information and then assembled into transcripts. Finally, singletons and the longest transcripts in each cluster were selected as a total reference Unigene set for sand rice. Histograms of Unigene length and GC content data were generated using an R script (R Development Core Team, 2008). Whole Unigenes were blasted against public protein databases, including the NCBI non-redundant protein (Nr) database, the Swiss-Prot protein database, Clusters of Orthologous Groups of proteins (COG), Gene Ontology (GO), Kyoto Encyclopedia of Genes and Genomes (KEGG), and Eukaryotic Orthologous Groups (KOG), using a threshold less than 1E-5. The predicted ORFs for each Unigene were aligned against the Pfam database using the program HMMER (Eddy, 1998) to increase the number of annotated Unigenes.

### SNP Calling and Statistical Analyses

Clean reads from each sample were re-mapped to reference Unigenes using the STAR program (Dobin et al., 2013) and SNP calling was conducted using GATK with the standard filter method (McKenna et al., 2010). Two main parameters were used to filter SNPs: (1) more than three mismatches in the adjacent 35 bp and (2) SNP quality score less than 2.0 after normalization for sequencing depth. This study mainly focused on pairwise SNPs, amongst which three types were classified based on their homozygous and heterozygous status in each sample. Confidently called SNPs were extracted from each Unigene and SNP frequencies were calculated by dividing the Unigene length by its number of SNPs. Histograms of SNP numbers and frequencies per Unigene, in addition to boxplots and ANOVA analysis of SNP frequency for each category, were performed using an R script (R Development Core Team, 2008).

For the GO enrichment analysis, a hypergeometric test with Bonferroni adjustment was used to identify enriched GO terms for Unigenes containing the pairwise SNPs, where the reference Unigene set served as the background. Terms were defined as enriched when adjusted *p*-values were less than 0.01.

### SNP Validation

Primers were designed to amplify fragments spanning one or two SNPs in 50 randomly selected Unigenes identified as containing pairwise SNPs by RNA-seq. Two DNA samples from individual SPT and NM plants served as templates for validation the predicted SNPs. ExTaq (TaKaRa, Dalian, China) was used to amplify the fragments with 1.5 μl template DNA in 20 μl reaction volume. The PCR reaction was performed in a C1000 TOUCH thermal cycler and the amplified products were sequenced using an ABI Prism 3730xl sequencer at Majorbio (Shanghai, China).

### Population Samples, Candidate Gene Sequencing, and Genetic Diversity Analyses

A total of 60 individuals from 10 natural populations (FK, DH, QHH, YJ, M4, MQ, SPT, JB, DLSH, NM) as described in Qian et al. (2016) were used to determine the genetic diversity of the three candidate genes (*HsfA1d, DAR1, LHY*, and *ELF3*). Samples from two individual plants from the SPT and NM populations were first amplified and sequenced in both directions to confirm the suitability of primers for population sequencing. PCR and sequencing were performed as described above. Population sequencing results for each candidate gene were aligned using BioEdit version 7.2.5 (Hall, 1999). After removing poor quality nucleotide sequence at the 5′ and 3′ termini, alignment files were subjected to further analysis of diversity indices using DnaSP version 5.10.01 (Librado and Rozas, 2009).

## Results

### Transcriptome Assembly and Annotation

**FIGURE 2:**
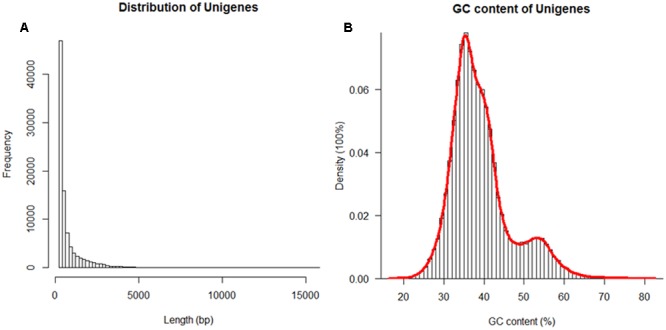
**Histograms of the length (A)** and the GC content of total assembled sand rice Unigenes. The red line in **(B)** indicates the density distribution.

An NM sand rice transcriptome library was constructed by mixing shoot and root RNA in a 1:1 ratio and pair-ended sequencing yielded 16.4 million clean reads. These reads were assembled, together with those from an individual sand rice plant from the SPT region (SRR1559276, Zhao et al., 2014) and a total of 91,884 Unigene were finally obtained with an average length of 725.98 bp and an N50 length of 1220 bp (**Figure 2A**). The average GC content was 39.14, and 69.36% of the total Unigenes had GC contents more than 35% (**Figure 2B**). All Unigenes were then blasted against six public protein databases, including the Nr, Swissprot, COG, GO, KOG, and KEGG, with a threshold of less than 1E-5. To increase the number of annotated Unigenes, predicted proteins were also aligned with the Pfam database. As shown in **Table 1**, 34,939 Unigenes (38.02%) could be matched to protein sequences or domains available in these databases.

**Table 1:** Summary of Unigenes annotation.

Annotation database	Annotated number	Length ≥ 300 bp	Length ≥ 1000 bp
COG	11,985	9,939	6,009
GO	18,973	15,037	8,244
KEGG	7,148	5,851	3,431
KOG	18,988	15,620	8,815
Pfam	21,641	18,910	12,138
SWISS	20,343	17,670	10,851
Nr	34,526	27,639	14,381
Total	34,939	27,841	14,403

### Pairwise SNP Detection, Statistical Analyses, and GO Annotation

**FIGURE 3:**
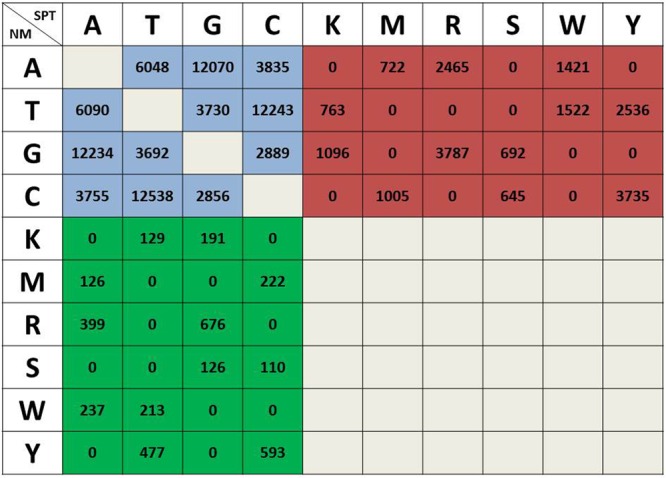
**Classification and statistical analysis of pairwise SNPs between sand rice individuals from the NM and SPT regions.** The three different background colors represent three types of pairwise SNPs between the two individuals and the number of each type of SNP is presented in each cell. Blue, inter-individual SNPs; green, NM specific SNPs; red, SPT specific SNPs. K, T/G; M, A/C; R, A/G; S, G/C; W, A/T; Y, T/C.

The clean reads from NM and SPT samples were re-mapped to the assembled reference Unigene set and SNPs were detected using GATK with default settings (McKenna et al., 2010). After quality filtering, a total of 111,971 and 119,702 SNPs were identified in NM and SPT individuals, respectively (Supplementary Table 1). The pairwised SNPs between NM and SPT were then isolated and classified into three types (**Figure 3**): 81,980 were inter-individual, which were homozygous in both samples; 3,499 were specific to NM, 20,389 to SPT, these were heterozygous in one individual but homozygous in the other. Fifty Unigenes were randomly selected for validation the predicted SNPs, of which 30 were successfully amplified and sequenced. The failed amplication might be from amplicons spanned a large intron or primers located at the exon/intron boundaries. Finally, 38 out of 50 SNPs were validated in these 30 Unigenes and another six were confirmed as homozygous SNPs. The predicting accuracy of our SNP dataset reached 76% (Supplementary Figure 2).

**FIGURE 4:**
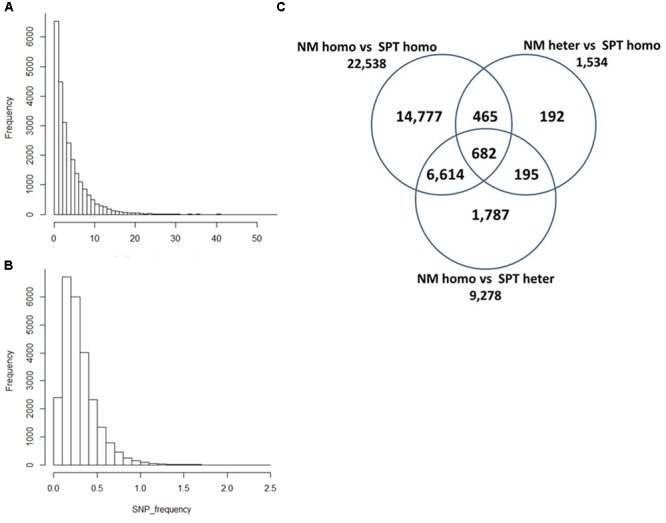
**Histograms of pairwise SNP number and frequency in sand rice transcriptomes and a Venn diagram of Unigenes containing different types of pairwise SNPs.** The SNP number **(A)** and frequency **(B)** per Unigene are presented. SNP frequency = Number of SNPs in each Unigene/corresponding Unigene length × 100%; **(C)** 24,712 Unigenes were classified into seven categories based on types of SNP in each Unigene. For example, 14,777 Unigenes contained only inter-individual SNPs and 682 Unigenes contained all three types of SNPs. The categories were named after the number of Unigenes in each category (e.g., SR_14777).

**FIGURE 5:**
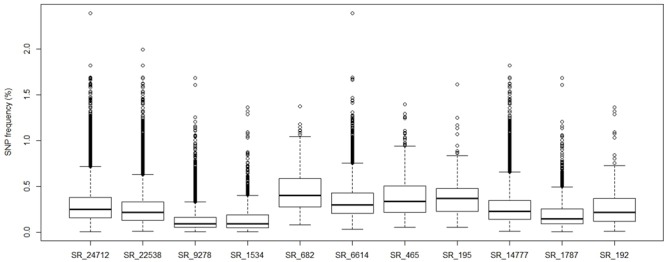
**Boxplot of pairwise sand rice SNP frequencies in each of the SNP defined Unigene categories described in **Figure 4****.

Single nucleotide polymorphism distribution and frequency are important indices for genome wide SNP development. In this study, all pairwise SNPs were distributed in 24,712 of 91,884 reference Unigenes, with approximately 57% (14,130) containing fewer than three SNPs (**Figure 4A**). A histogram of SNP frequency revealed a peak at 0.2 and 61.24% Unigenes were under the average SNP frequency of 0.3% (**Figure 4B**). Among the 24,712 Unigenes, 22,538 contained inter-individual SNPs, and 1,534 and 9,278 contained NM- and SPT-specific SNPs, respectively (**Figure 4C**). There were 16,756 Unigenes containing only one type of SNP, while 682 had all three types. Categories were named according to the gene number in each set in the Venn diagram (**Figure 5**). The mean SNP frequency values for all categories were lower than 0.45%; however, the four categories (SR_682, SR_6614, SR_465, and SR_195) including two or three types of SNP, exhibited higher frequencies than the other categories (*p* < 0.01; **Figure 5**). Detailed histograms illustrating SNP numbers and frequencies for each category are presented in Supplementary Figure 3.

Genes in each category (**Figure 4C**) were separately subjected to GO enrichment analysis (**Table 2** and Supplementary Table 2). For the whole Unigene set (SR_24712), the most enriched GO terms were related to basic biology processes, including ‘DNA integration,’ ‘RNA-dependent DNA replication,’ and ‘translation.’ The GO enrichment results for inter-individual category (SR_22538) were similar to those for SR_24712, except they also included ‘translation elongation’ (GO: 0006414, *p* = 0.04). The GO term, ‘DNA recombination’ (GO: 0006310) was enriched for NM-specific category (SR_1534, *p* = 0.02) and not for any of the other categories. Interestingly, the GO term, ‘thylakoid membrane organization’ (GO: 0010027), was found in the enrichment results of SPT-specific category (SR_9278) and SR_24712, suggesting genes in SR_6614 were largely responsible for this annotation (*p* = 0.03). SR_14777 included only genes containing inter-individual SNPs (**Figure 4C**) and was enriched for seven GO terms, including ‘response to misfolded protein’ (GO: 0051788) and ‘response to cold’ (GO: 0009409). Of note, the GO term ‘embryo development ending in seed dormancy’ (GO: 0009793) was also enriched in categories SR_24712, SR_22538, and SR_14777.

**Table 2:** Gene ontology enrichment results of SR_24712 and SR_14777 categories.

GO ID	GO term	*p*-values	adj-*p*-values
**SR_24712**			
GO:0015074	DNA integration	8.00E-17	1.61E-13
GO:0006278	RNA-dependent DNA replication	1.27E-11	2.54E-08
GO:0006412	Translation	2.28E-09	4.57E-06
GO:0055085	Transmembrane transport	2.60E-08	5.20E-05
GO:0009793	Embryo development ending in seed dormancy	3.17E-07	0.000635
GO:0019288	Isopentenyl diphosphate biosynthetic process, methylerythritol 4-phosphate pathway	7.49E-07	0.001498
GO:0043581	Mycelium development	2.17E-06	0.004338
GO:0006357	Regulation of transcription from RNA polymerase II promoter	3.71E-06	0.007416
GO:0006259	DNA metabolic process	5.39E-06	0.010762
GO:0010027	Thylakoid membrane organization	2.09E-05	0.041638
**SR_14777**			
GO:0015074	DNA integration	4.30E-15	6.53E-12
GO:0006278	RNA-dependent DNA replication	3.26E-09	4.94E-06
GO:0051788	Response to misfolded protein	8.55E-07	0.001297
GO:0055085	Transmembrane transport	2.96E-06	0.00449
GO:0009793	Embryo development ending in seed dormancy	6.57E-06	0.00994
GO:0009409	Response to cold	1.00E-05	0.015188
GO:0006412	Translation	1.29E-05	0.019432

### Candidate Genes Relevant to Heat Tolerance and Agronomic Traits

**FIGURE 6:**
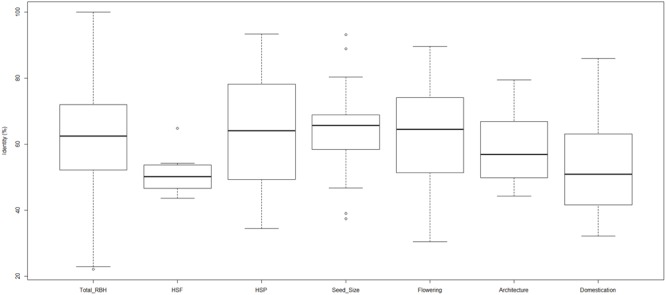
**Boxplot of sequence identity values for total reciprocal best hits (RBHs), heat-shock transcription factor (HSF), heat shock protein (HSP), seed size, flowering, architecture, and domestication proteins between *Arabidopsis* and sand rice**.

To isolate candidate genes for heat tolerance and agronomic traits, an all-against-all blast approach was conducted (described in Zhao et al., 2014) between the assembled sand rice Unigenes and the *Arabidopsis* protein database, resulting in the identification of 10,512 pairs of reciprocal best hits (RBHs) (**Figure 6** and Supplementary Table 3). A Literature survey identified 64 heat shock proteins (HSPs), 21 HSFs, 366 flowering-, 59 seed size-, and 21 architecture-related genes in *Arabidopsis* (Finka et al., 2011; Scharf et al., 2012; Kesavan et al., 2013; Xiao et al., 2013; Dong and Wang, 2015; Li and Li, 2015, 2016; Teichmann and Muhr, 2015) and 31 HSPs, 11 HSFs, 100 flowering-, 28 seed size-, and 11 architecture-related genes had RBHs in the sand rice transcriptome, with mean sequence identities ranging from 50.88 to 64.09% (**Figure 6** and Supplementary Table 3). Notably, only two Hsf1A sequences were identified in sand rice. Coincidently, the DA1 was missing from sand rice, while DAR1 was present. These lines of evidence imply that the gene families encoding these proteins may not have undergone expansion during sand rice speciation (Zhao et al., 2014). Furthermore, 41 genes with clear functions in the domestication of crop plants, including teosinte branched 1 in maize (Doebley et al., 1997), ring-type E3 ubiquitin ligase in rice (GW2; Song et al., 2007), non-brittle rachis 1 and 2 in barley (Pourkheirandish et al., 2015), tannin1 in sorghum (Wu et al., 2012), were blasted against the sand rice Unigene set, resulting in the identification of 39 RBHs with an average sequence identity of 53.78% (**Figure 5** and Supplementary Table 3). By comparison with SR_24712 category, 30 HSPs, 11 HSFs, 96 flowering-, 26 seed size-, 11 architecture-, and 32 domestication-related genes containing at least one type of SNPs, were identified (Supplementary Table 3).

### Genetic Diversity of *HsfA1d, DAR1, LHY*, and *ELF3* in Natural Populations

**FIGURE 7:**
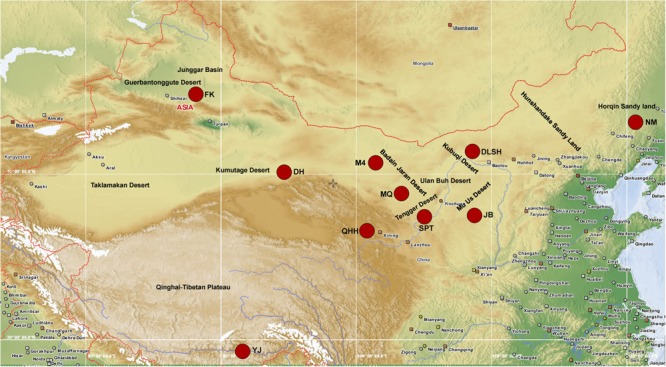
**Sampling sites**. This map was generated using PopART-1.7 (Bandelt et al., 1999) based on the latitude and longitude of each population.

**FIGURE 8:**
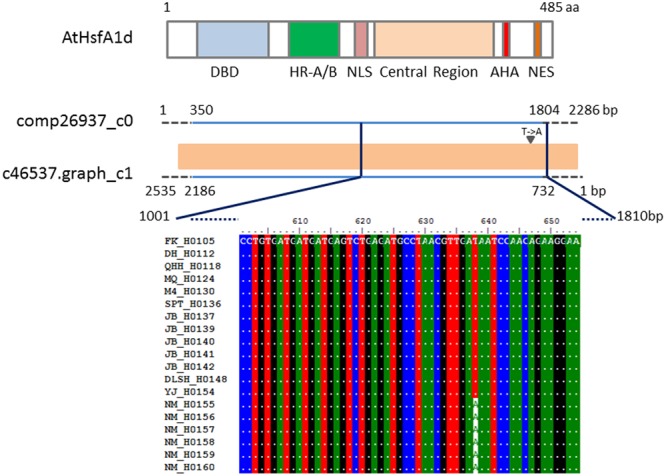
**Schematic of *Arabidopsis* and sand rice HsfA1d protein structure and multiple sequence alignment results.** The *Arabidopsis* HsfA1d protein (AtHsfA1d) structure is adapted from Ohama et al. (2015); the DNA-binding domain (DBD), oligomerization domain (HR-A/B), nuclear localization signal (NLS), transactivation domain (AHA), and nuclear export signal (NES) are indicated. Based on the RBH results, one previously assembled Unigene sequence (comp26937_c0, 2286 bp; Zhao et al., 2014) and one Unigene identified in this study (c46537.graph_c1, 2535 bp) are indicated by lines. The orange rectangle represents a region of identity between the two Unigenes. Sky-blue lines represent similar regions (350–1804 bp in comp26937_c0 and 2186–732 bp in c46537.graph_c1) between the AtHsfa1d protein and sand rice Unigenes. The vertical dark blue lines indicate the fragment (1001–1810 bp), that was amplified from sand rice samples from 10 natural populations. The multiple sequence alignment includes all of the successful sequencing results for the JB and NM populations, along with representative sequences from each of the eight other populations. The arrowhead indicates the T/A SNP.

Phylogeographic analysis has revealed that sand rice originated from the Gurbantunggut desert and then dispersed into the central desert region and eastern sandy lands in China (Qian et al., 2016). In this study, 10 natural populations (**Figure 7**) from the Gurbantunggut desert (FK), Kumutage desert (DH), Qinghai-Tibetan Plateau (QHH and YJ), central deserts (M4, MQ, SPT, JB, DLSH), and eastern sandy regions (NM), were used to investigate the genetic diversity of four candidate genes. Six genotypes in each population were sequenced and the successful genotypes for each gene ranged from 51 to 54 (**Table 3**). The *HsfA1d* gene was conserved in nine populations, with a single transversion SNP (T/A) leading to an amino acid change (Glutamate/Aspartate) detected in NM population (**Figure 8**). Alignment based on the RBH results demonstrated that this SNP was located between the regions encoding the transactivation domain (AHA) and the nuclear export signal (NES) at the C terminus of *Arabidopsis* HsfA1d. For the seed size gene *DAR1*, an intron (125 bp) was included in the PCR products and population sequencing identified a single transversion SNP (G/C), specific to the NM population. This synonymous SNP encoded an amino acid located between the LIM and the LIM-associated C-terminal domains (Supplementary Figure 4). Similarly, an 86 bp intron was included in the *LHY* amplicons and four SNPs were obtained from across the 10 sand rice populations, of which three were transitions (T/C, C/T, and T/C) and one was a transversion (A/T) leading to an amino acid change (Glutamine/Histidine; Supplementary Figure 5). Two transition SNPs were detected in the *ELF3* gene. One SNP (G/A) was NM-specific and synonymous, while the other (C/T) was observed in five of six genotypes in the JB population and encode an Alanine to Valine amino acid substitution (Supplementary Figure 6). The aligned sequences for each gene were further subjected to analysis of nucleotide and haplotype diversity using DnaSP. Consistent with the numbers of SNP, nucleotide diversity values for all three genes were very low, ranging from 0.00032 (*HsfA1d*) to 0.00118 (*LHY*), while haplotype diversity ranged from 0.208 (*HsfA1d*) to 0.352 (*ELF3*).

**Table 3:** Nucleotide and haplotype diversity of four genes in 10 natural populations.

Candidate gene	*HsfA1d*	*DAR1*	*ELF3*	*LHY*
Expected length (bp)	810	539	699	866
PCR length (bp)	810	664	699	952
Sequence length (bp)	654	459	626	849
No. of genotypes	52	51	54	53
No. of indels	0	0	0	0
Indel frequency	0	0	0	0
No. of SNPs	1	1	2	4
Transition	0	0	2	3
Transversion	1	1	0	1
SNP frequency	1/654	1/459	1/313	1/212
Nucleotide diversity (Pi)	0.00032	0.00046	0.00059	0.00118
Watterson’s parameter (𝜃_w_)	0.00034	0.00048	0.0007	0.0009
No. of haplotypes	2	2	3	3
Haplotype diversity	0.208	0.212	0.352	0.312

## Discussion

Dissection of the genetic variation of sand rice is essential for understanding its extreme stress tolerance and phenotypic variability and will facilitate its future domestication. In this study, a total of 105,868 pairwise SNPs, distributed in 24,712 Unigenes, were identified between SPT and NM samples, with an average SNP frequency of 0.3% (**Figures 2**, **3**). There were a larger number of SNPs specific to SPT (81,980 in 9,278 Unigenes) than to NM (3,499 in 1,534 Unigenes), implying that sand rice in the SPT region may exhibit a higher degree of outcrossing, and, consequently, increased genomic heterozygosity. GO enrichment analysis identified only one term (GO: 0006310, ‘DNA recombination’) significantly enriched for SR_1534 (**Table 2**), suggesting that variations in specific genes were required for the survival of the NM population in the semi-arid environment.

Environmental stresses, such as drought, heat, and salinity, adversely affect plant photosynthesis (Nouri et al., 2015). The thylakoid membrane is the primary site of photosynthesis inside chloroplasts and its molecular organization is vital for the coordination and regulation of photosynthetic processes (Nouri et al., 2015). Given that SPT is an arid, desert environment, it is logical that the GO term ‘thylakoid membrane organization’ is enriched in the SPT specific category (SR_9278). Two terms associated with the response to misfolded protein (GO: 0051788) and cold (GO: 0009409) were identified as enriched in the inter-individual category (SR_14777). These results may provide evidence that common genetic modules are shared in natural populations, whereas different alleles are favored by direction selection pressure resulting from the prevailing environmental conditions in the NM and SPT regions. Another abundant term in SR_14777 was ‘embryo development ending in seed dormancy’ (GO: 0009793), which is consistent with a survival strategy to cope with sand bury and unpredictable precipitation typical in the geographic range of sand rice (Tobe et al., 2005; Zheng et al., 2005; Liu et al., 2006; Gao et al., 2014).

In *Arabidopsis*, numerous genes have been identified with important roles in stress responses and controlling agronomic traits, such as large seed size, plant architecture, and earlier flowering. In this study, 10,512 pairs of orthologous genes were identified between *Arabidopsis* and sand rice (Supplementary Table 3). Among these, c48914.graph_c0 was included in the top 50 Unigenes with the highest numbers of SNPs; the ortholog in *Arabidopsis* was maintenance of methylation 1 (*MOM1*), which is involved in silencing the stress-induced expression of transposons and thereby in preventing the transgenerational transmission of epigenetic memory (Iwasaki and Paszkowski, 2014). It is possible that different alleles of *MOM1* may have evolved in the NM and SPT sand rice populations to control epigenetic stress memory in the face of reoccurring heat- and other types of stress-induced damages. A number of orthologous genes in the sand rice transcriptome dataset that are candidates for involvement in the heat stress response and the control of agronomic traits are also listed in Supplementary Table 3, and the majority of them contained sequence variation between NM and SPT. Dissecting the genetic diversity of these candidate genes and analyzing their association with phenotypic variability in natural populations is likely to result in the identification of important molecular markers and/or favorable alleles, and to facilitate the domestication process of sand rice.

The sequence identity value (54%) between *Arabidopsis HsfA1d* and sand rice c46537.graph_c1 suggests that these genes may encode functionally equivalent proteins involved in heat stress tolerance. Quantitative RT-PCR revealed that the expression pattern of c46537.graph_c1 was similar to that of *Arabidopsis HsfA1d* after heat stress treatment (Supplementary Figure 7). A fragment of c46537.graph_c1 was analyzed in 10 natural populations, resulting in the identification of a single unique allele in the NM population. Similarly, only one synonymous SNP was detected in sand rice *DAR1* by population sequencing. These results demonstrate that the same *HsfA1d* and *DAR1* haplotypes were shared by plants in the central deserts and that genetic divergence has mainly occurred between NM and the other populations. Both of the SNPs in *HsfA1d* and *DAR1* located in sequences encoding inter-domain protein regions, hence it is difficult to predict the effect of these SNPs on sand rice adaptation based on knowledge of protein domain functions determined in *Arabidopsis*. The non-synonymous SNP in *HsfA1d* may compromise the trimer formation (HsfA1a, b, and d), resulting in ineffective activation of the transcription network, which would be consistent with the reduced heat tolerance phenotype associated with NM plants (Zhao et al., 2014).

*LHY* and *ELF3* are important regulators of the circadian rhythm (Hicks et al., 1996; Nusinow et al., 2011; Hsu and Harmer, 2014). The eastern sandy population (NM) harbored specific *LHY* and *ELF3* alleles which were separated from other populations. These results suggest that variants in these two genes could confer some fitness advantage, such as early flowering, in the semi-arid environment. Furthermore, two transition SNPs of *LHY*, were shared between JB and NM populations. This is congruent with the speculation that the sand rice colonization pathway originated from the Gurbantunggut desert and subsequently dispersed into other desert regions (Qian et al., 2016).

## Author Contributions

PZ conceived and designed the project. JZ prepared the RNA for sequencing. CQ and X-FM supplied the population DNA samples. QZ and XZ helped to perform experiments. PZ conducted the experiments, analyzed the results, and wrote the manuscript and GC revised the manuscript.

## Conflict of Interest Statement

The authors declare that the research was conducted in the absence of any commercial or financial relationships that could be construed as a potential conflict of interest.
