# Supplementary material for: SNP Discovery and Genetic Variation of Candidate Genes Relevant to Heat Tolerance and Agronomic Traits in Natural Populations of Sand Rice (Agriophyllum squarrosum)
Source: Front Plant Sci. 2017 Apr 7;8:536. doi: 10.3389/fpls.2017.00536 (PMC5383723; doi:10.3389/fpls.2017.00536)
Supplement: Supplementary file 1 [file Data_Sheet_1.docx]

Supplementary Material

SNP discovery and genetic variation of candidate genes associated with heat tolerance and agronomic traits in natural populations of sand rice (Agriophyllum squarrosum)

Pengshan Zhao^1,2^*, Jiwei Zhang^1^, Chaoju Qian^1^, Qin Zhou^1^, Xin Zhao^1,2^, Guoxiong Chen^1,2^, Xiao-Fei Ma^1^

*** Correspondence:** Pengshan Zhao: zhaopengshan@lzb.ac.cn

# Supplementary Data

Raw data of this article were deposited in the NCBI Short Read Archive with the accession number SRR5271162.

# Supplementary Figures and Tables

## Supplementary Figures

**
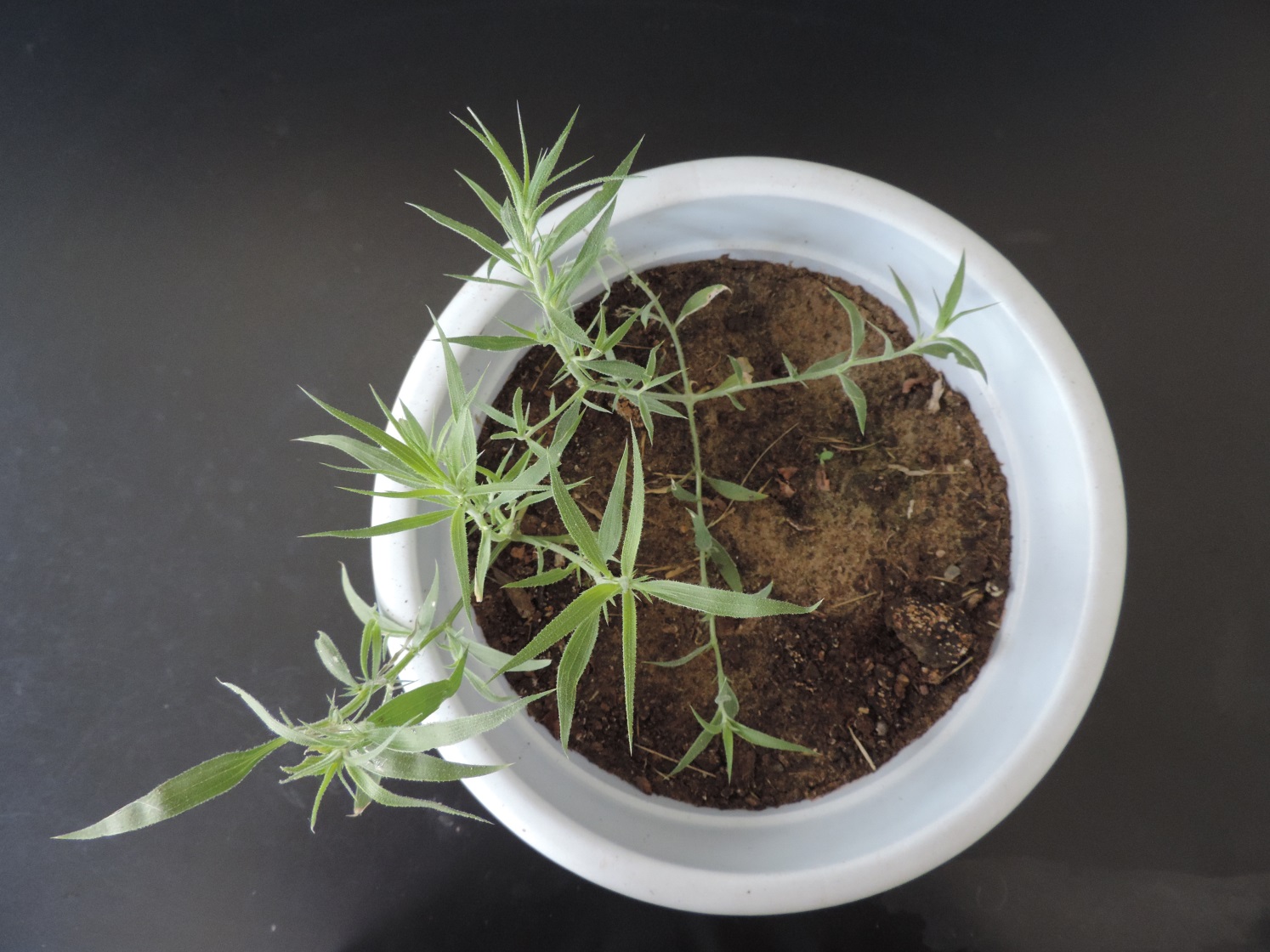
**

**Supplementary Figure 1.** Sand rice plant used for RNA-seq in this study.


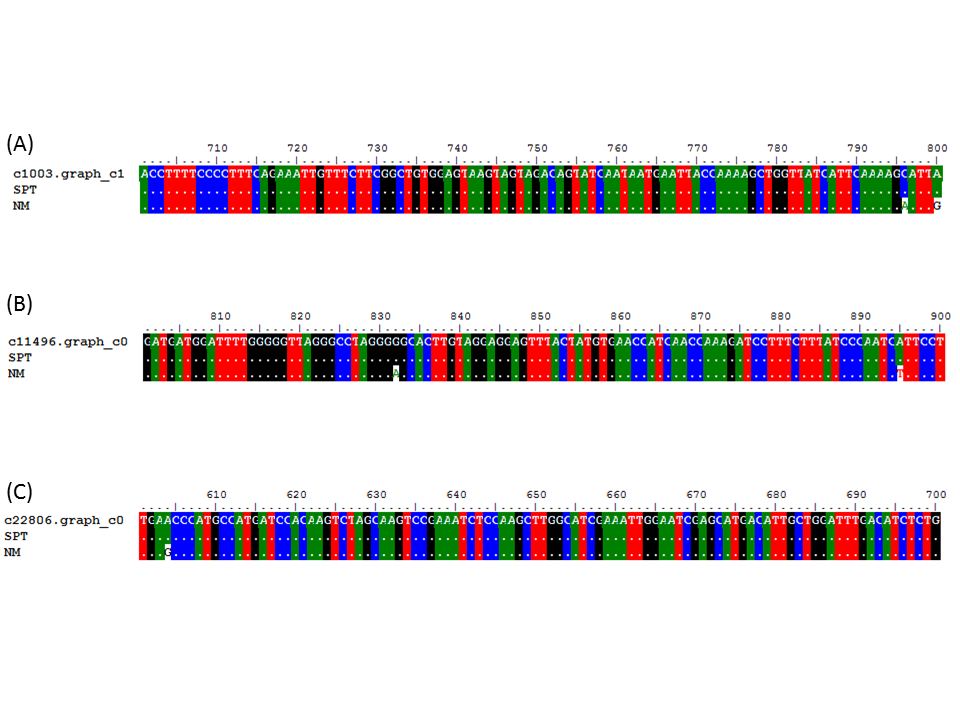


**Supplementary Figure 2.** Validation of predicted pairwise sand rice SNPs. **(A-C)** Three representative alignment results are shown. One genotype from each population was aligned with the assembled Unigene. For example, c1003.graph_c1 (A) is the assembled Unigene in this study. SPT represents the sequencing result of the genotype from the SPT population and NM is the sequencing result of the genotype from the NM population.

**Data sheet 2**

**Supplementary Figure 3.** Histograms of pairwise SNP number and frequency for sand rice Unigenes in different categories (as defined in Figure 4c).

**
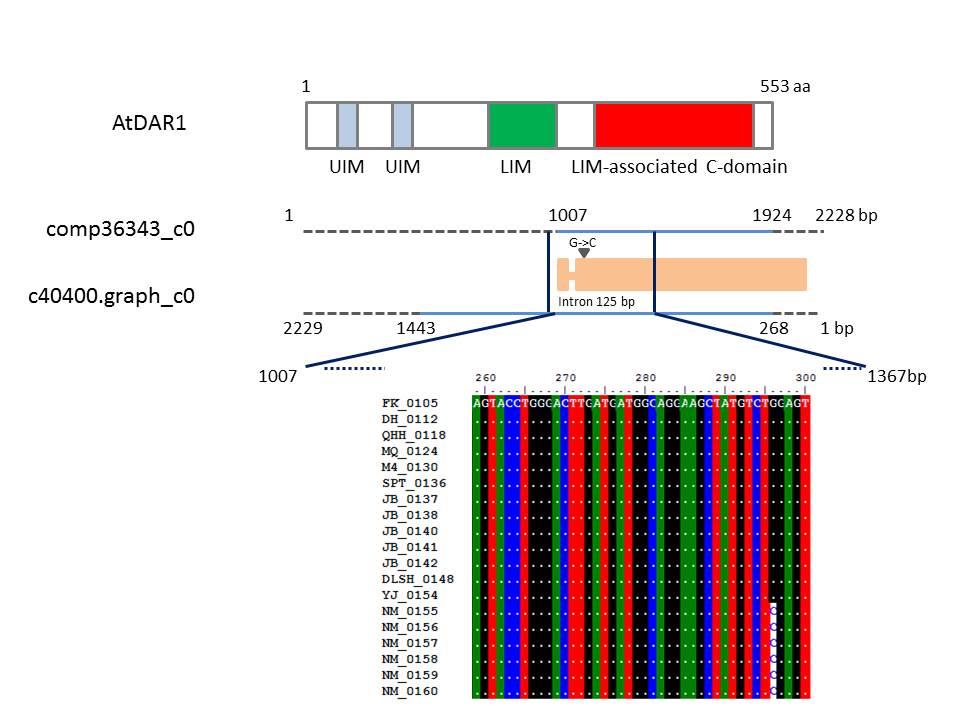
**

**Supplementary Figure 4.** Schematic of the *Arabidopsis* and sand rice DAR1 proteins and multiple sequence alignment results. The *Arabidopsis* DAR1 protein (AtDAR1) structure is adapted from Zhao et al. (Zhao et al., 2014a); two ubiquitin interaction motifs (UIM), a LIM domain, and a LIM associated and conserved C-domain are indicated. A previously assembled Unigene (comp36343_c0, 2228 bp; (Zhao et al., 2014b)) and a Unigene identified in this study (c40400.graph_c0, 2229 bp) are presented in detail, as described in Figure 7. The 5’ 132 bp of the PCR product showed no similarity between the two Unigenes and was, therefore, removed from the final alignment results. The single G/C SNP is indicated by as an arrowhead.


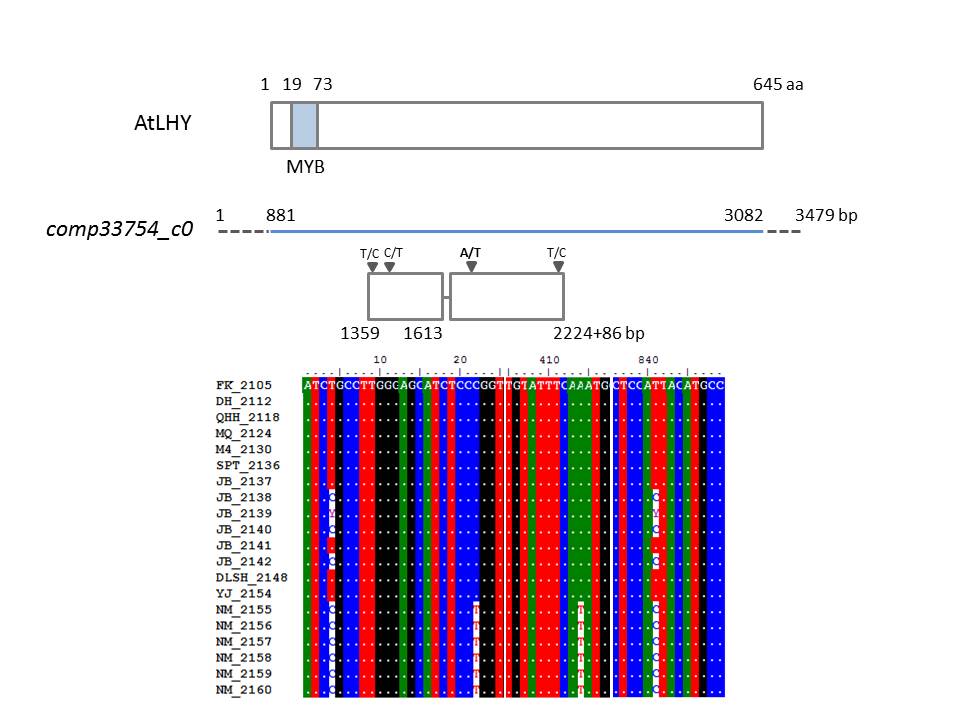


**Supplementary Figure 5.** Schematic of *Arabidopsis* LHY protein structure, and multiple sequence alignment results of the sand rice *LHY* gene. The *Arabidopsis* LHY (AtLHY) protein structure is shown, based on the description by TAIR10 (http://www.arabidopsis.org/), and a MYB domain is indicated at the N-terminal. The sand rice *LHY* orthologous unigene (comp33754_c0) is indicated by a line, and the sky-blue line represents the region (881–3082 bp) with similarity to the *Arabidopsis* LHY peptide. The unigene fragment from 1359 to 2224 bp, which contained an 86 bp intron, was amplified across ten natural populations with a total of 60 genotypes. Four SNPs are shown above arrowheads, and the transversion SNP is highlighted in bold type. The multiple sequence alignment includes all of the successful sequencing results for the JB and NM populations, along with representative sequences from each of the eight other populations.


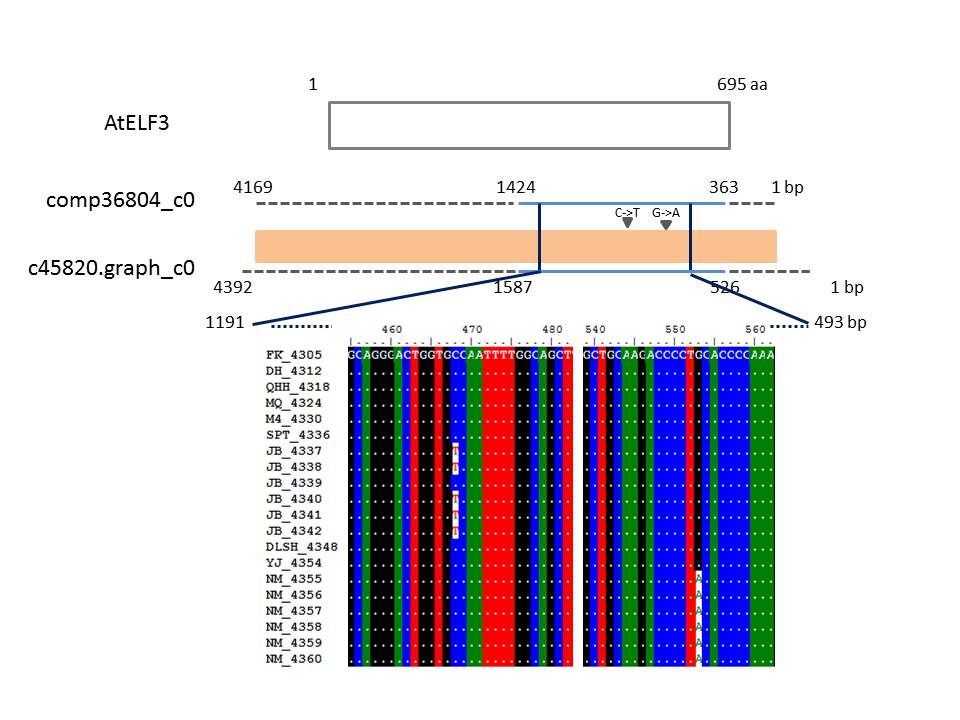


**Supplementary Figure 6.** *Arabidopsis* and sand rice ELF3 proteins and multiple sequence alignment results. The *Arabidopsis* ELF3 protein (AtELF3) does not contain any conserved protein domains. The previously assembled Unigene (comp36804_c0, 4169 bp; (Zhao et al., 2014b)) and the Unigene determined in this study (c45820.graph_c0, 4392 bp) are presented in detail, as described in Figure 7. Two population-specific SNPs (C/T in JB and G/A in NM) were identified by population sequencing.


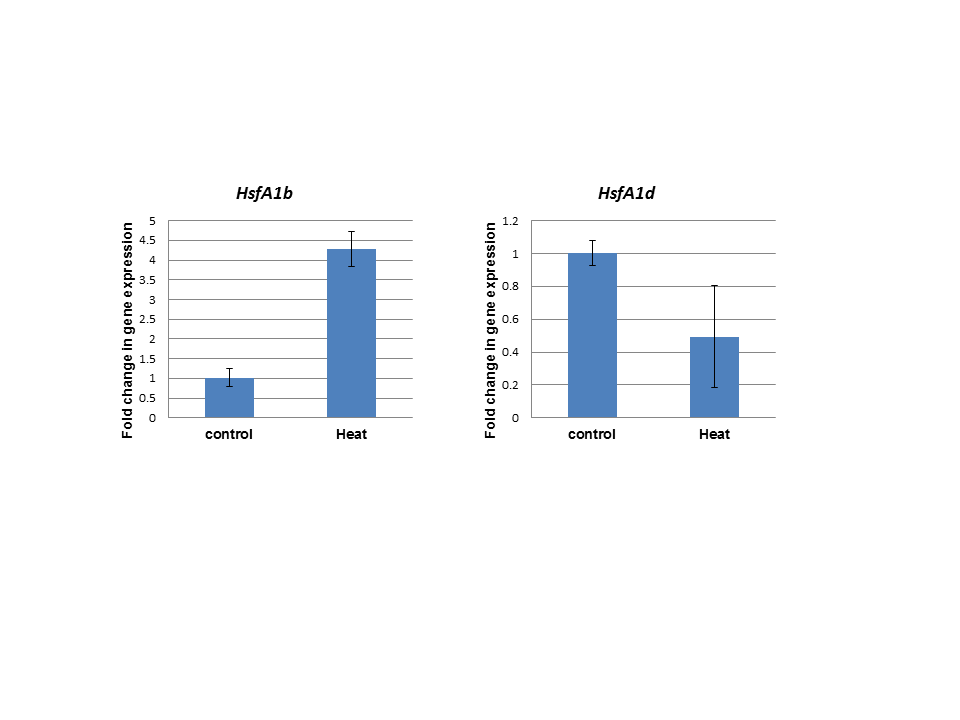


**Supplementary Figure 7.** Validation of sand rice *HsfA1b* and *HsfA1d* by RT-PCR. Total RNA was isolated from normal or heat treated (55°C for 3 hours) leaves. The internal reference gene was comp41623_c0 (*Elongation factor 1*). Each RNA sample was assayed in triplicates and two independently biological repeats were conducted.

## Supplementary Tables

**Supplementary Table 1.** Summary of SNPs in sand rice individuals from the NM and SPT regions.

| Samples | Homozygous SNP | Heterzygous SNP | Total |
| --- | --- | --- | --- |
| NM | 103,903 | 8,068 | 111,973 |
| SPT | 87,304 | 32,398 | 119,702 |

**Supplementary Table 2.** Summary of GO enrichment analyses of Unigenes from different categories (as defined in Figure 4c).

| GO ID | GO Term | p values | adj-p values |
| --- | --- | --- | --- |
| SR_22538 |  |  |  |
| GO:0015074 | DNA integration | 2.47E-19 | 4.75E-16 |
| GO:0006278 | RNA-dependent DNA replication | 5.92E-13 | 1.14E-09 |
| GO:0006412 | translation | 4.17E-12 | 8.00E-09 |
| GO:0009793 | embryo development ending in seed dormancy | 1.75E-07 | 0.000336 |
| GO:0055085 | transmembrane transport | 2.66E-07 | 0.00051 |
| GO:0043581 | mycelium development | 3.24E-07 | 0.000621 |
| GO:0019288 | isopentenyl diphosphate biosynthetic process, methylerythritol 4-phosphate pathway | 4.58E-07 | 0.000877 |
| GO:0006259 | DNA metabolic process | 7.13E-07 | 0.001364 |
| GO:0006357 | regulation of transcription from RNA polymerase II promoter | 1.04E-05 | 0.019989 |
| GO:0006414 | translational elongation | 2.17E-05 | 0.04152 |
|  |  |  |  |
| SR_9278 |  |  |  |
| GO:0015074 | DNA integration | 1.15E-07 | 0.000177 |
| GO:0006412 | translation | 1.23E-06 | 0.001896 |
| GO:0006278 | RNA-dependent DNA replication | 1.22E-05 | 0.018757 |
| GO:0010027 | thylakoid membrane organization | 1.76E-05 | 0.027025 |
| GO:0019288 | isopentenyl diphosphate biosynthetic process, methylerythritol 4-phosphate pathway | 2.55E-05 | 0.039294 |
|  |  |  |  |
| SR_1534 |  |  |  |
| GO:0006310 | DNA recombination | 3.01E-05 | 0.015988 |
|  |  |  |  |
| SR_6614 |  |  |  |
| GO:0015074 | DNA integration | 1.42E-14 | 1.97E-11 |
| GO:0006412 | translation | 7.76E-09 | 1.07E-05 |
| GO:0006259 | DNA metabolic process | 7.73E-08 | 0.000107 |
| GO:0006278 | RNA-dependent DNA replication | 1.20E-07 | 0.000166 |
| GO:0019288 | isopentenyl diphosphate biosynthetic process, methylerythritol 4-phosphate pathway | 8.86E-06 | 0.012191 |
| GO:0010027 | thylakoid membrane organization | 2.07E-05 | 0.028468 |
| GO:0016558 | protein import into peroxisome matrix | 2.63E-05 | 0.036183 |

**Supplementary Table 3.** Summary of protein Blast results, as presented in Figure 6. Unigenes containing SNP are highlighted in green.

Data sheet 3
