# Supplementary material for: SNP Discovery and Genetic Variation of Candidate Genes Relevant to Heat Tolerance and Agronomic Traits in Natural Populations of Sand Rice (Agriophyllum squarrosum)
Source: Front Plant Sci. 2017 Apr 7;8:536. doi: 10.3389/fpls.2017.00536 (PMC5383723; doi:10.3389/fpls.2017.00536)

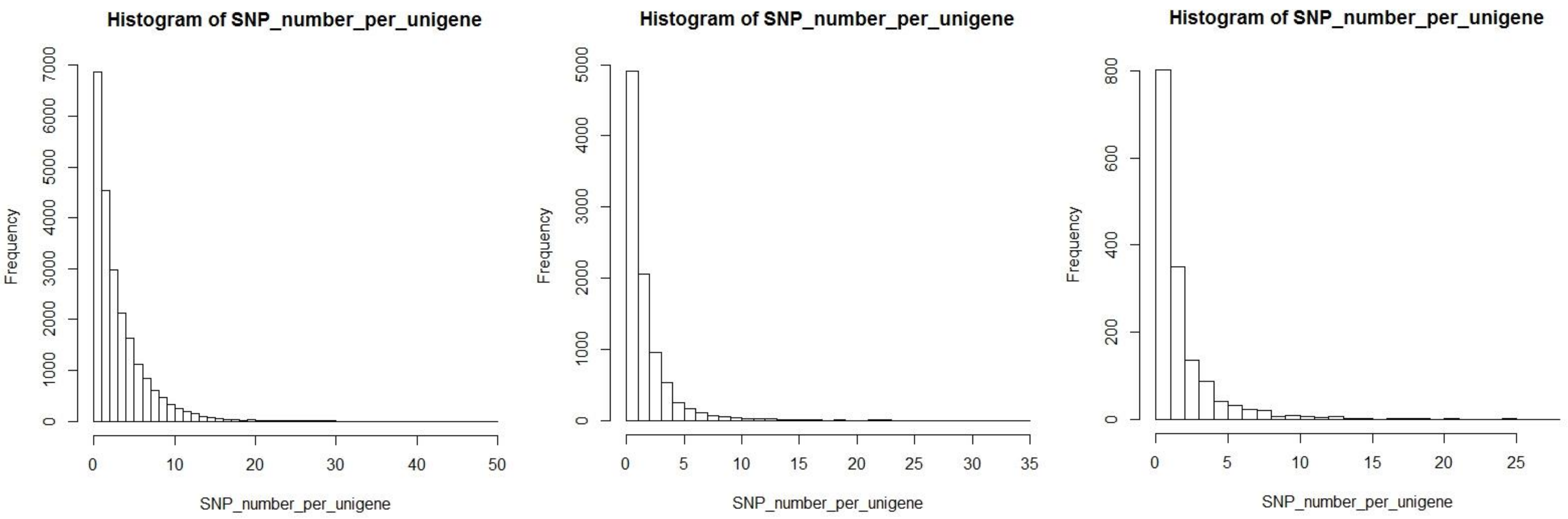

SR_22538 SR_9278 SR_1534

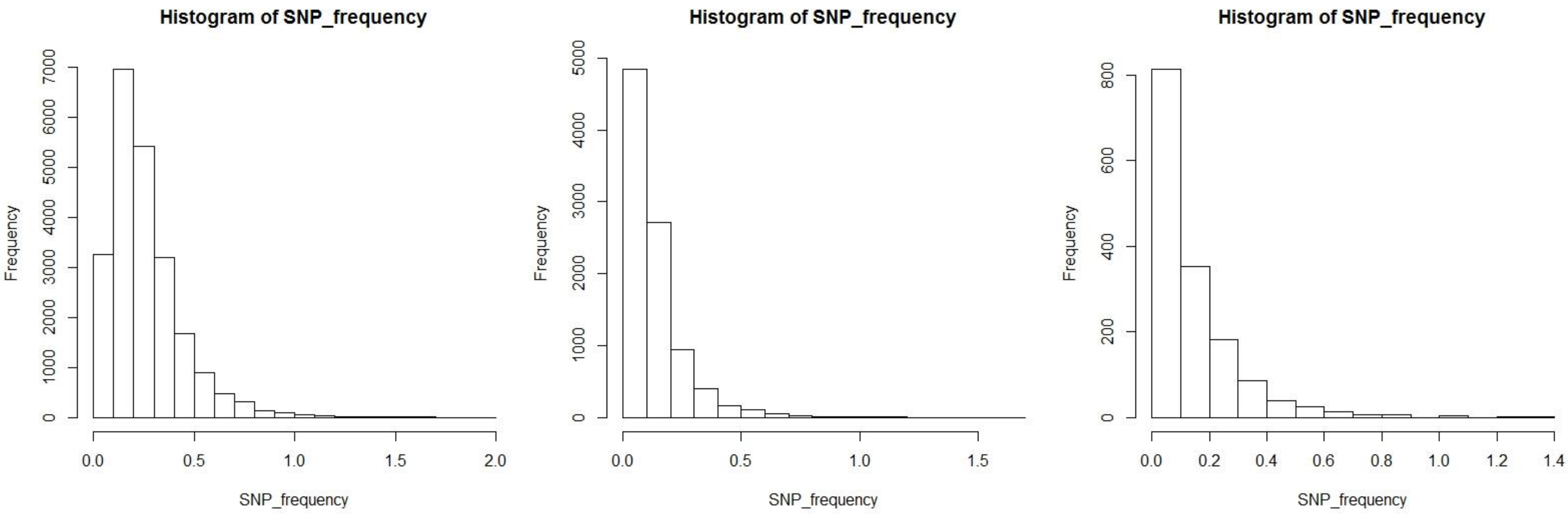

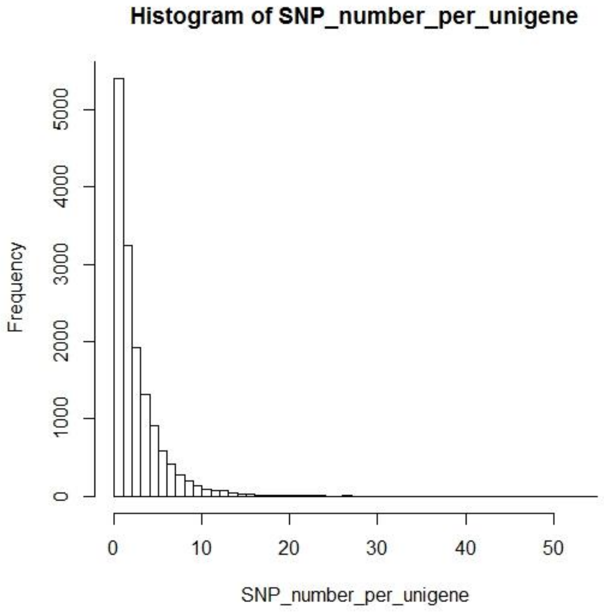

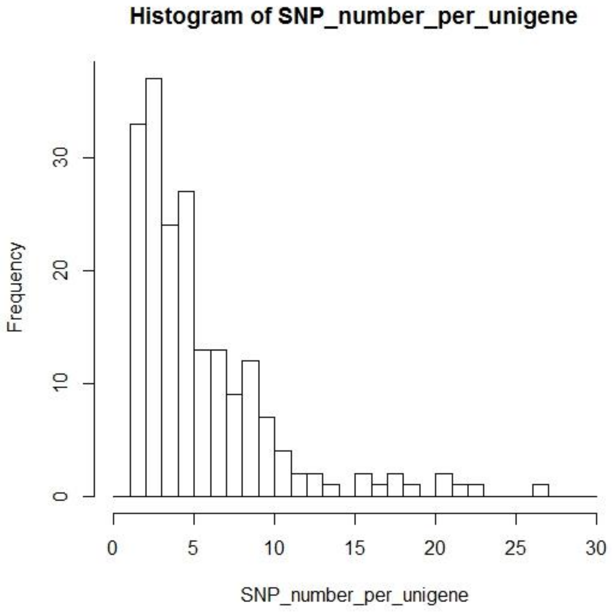

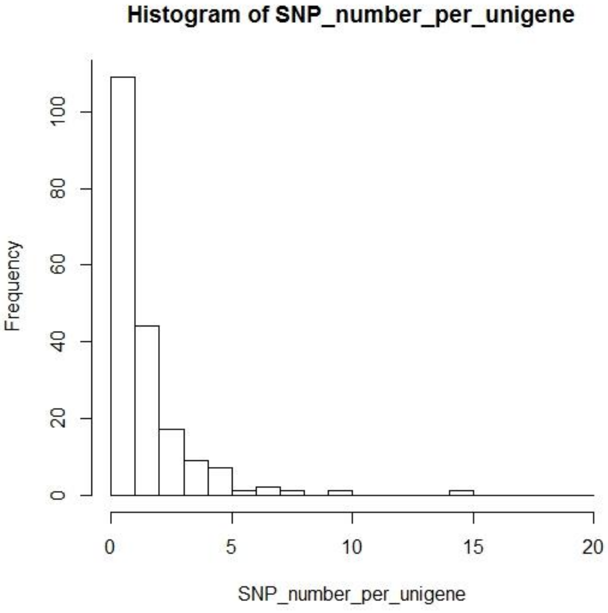

SR_14777

SR_1787

SR_192

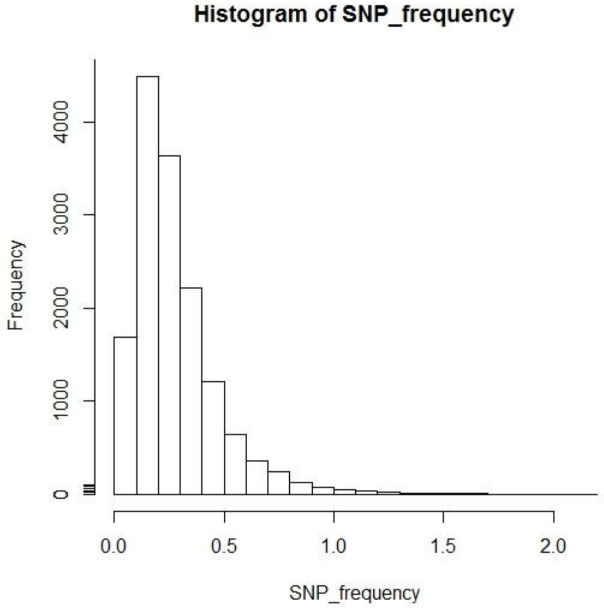

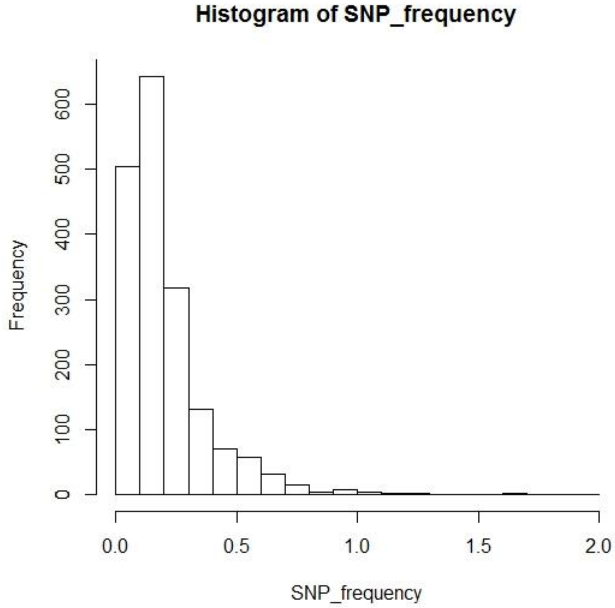

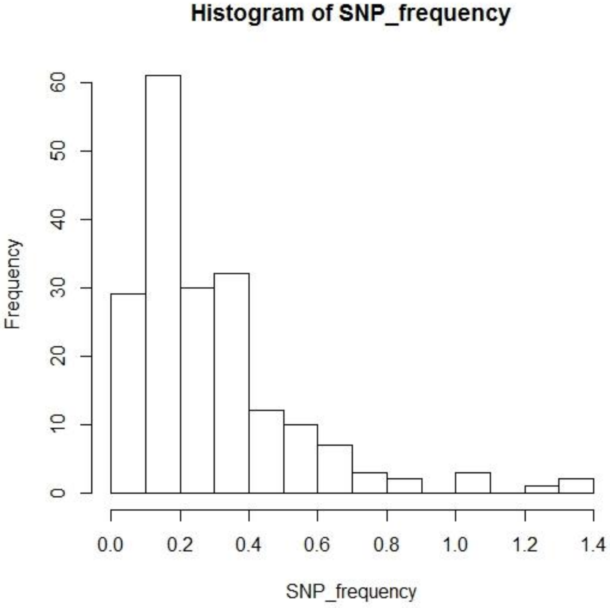

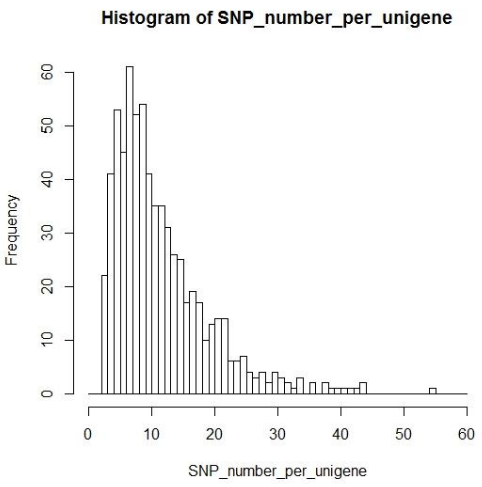

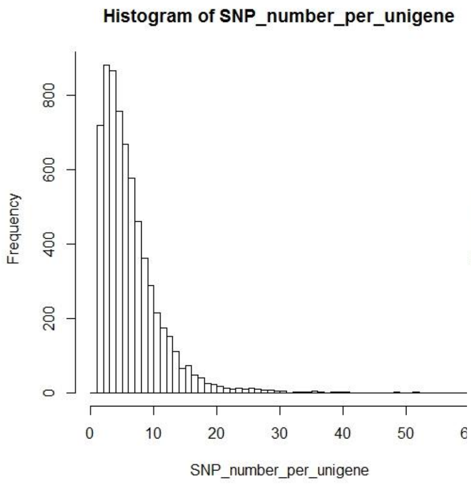

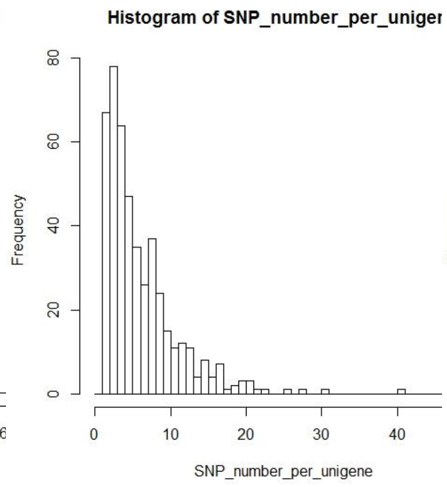

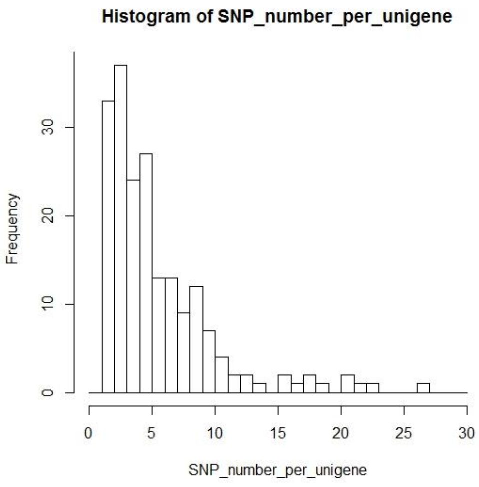

SR_682

SR_6614

SR_465

SR_195

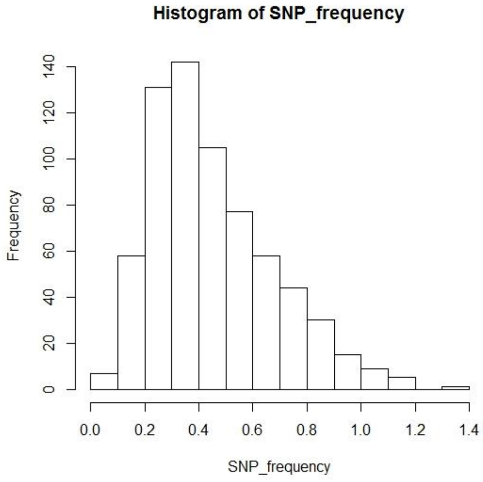

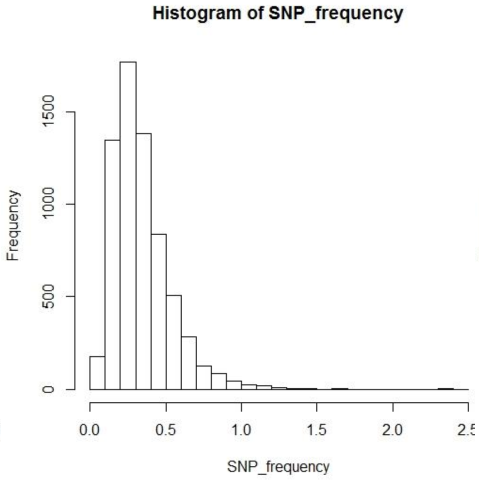

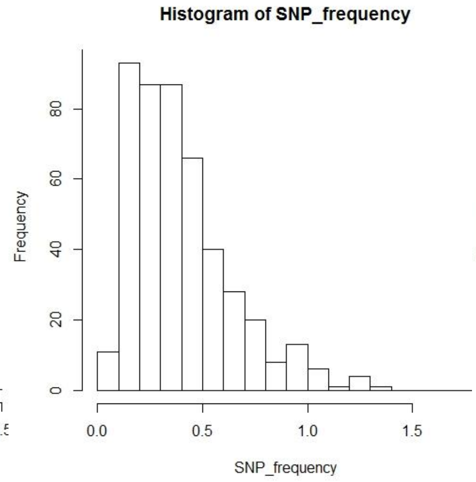

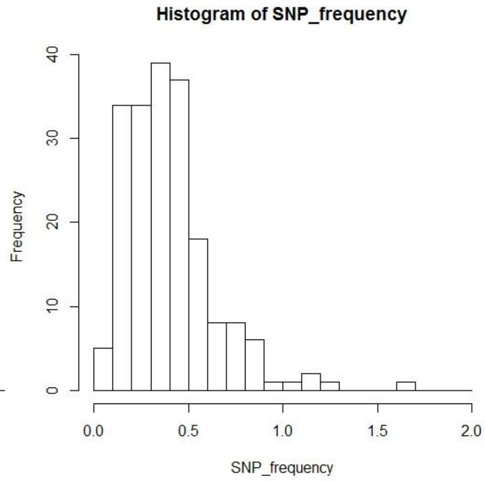

Supplement: Supplementary file 2 [file Data_Sheet_2.pdf]
